# Supplementary material for: Cost-effectiveness of incorporating Ebola prediction score tools and rapid diagnostic tests into a screening algorithm: A decision analytic model
Source: PLoS One. 2023 Oct 17;18(10):e0293077. doi: 10.1371/journal.pone.0293077 (PMC10581462; doi:10.1371/journal.pone.0293077)
Supplement: S1 File — (DOCX) [file pone.0293077.s001.docx]

**Detailed description of algorithms tested in the decision tree model**

| **Algorithm** | **Screening test used** | **Details description** |
| --- | --- | --- |
| Algorithm 1 | WHO case definition for EVD suspect used alone | “*Any person alive or dead, living in epidemic areas or any person who traveled to these areas during this period and who reported the following signs or symptoms; sudden fever onset and at least three of the following symptoms: vomiting, diarrhea, abdominal pain, conjunctivitis, rash, unexplained bleeding from any part of the body, muscle pain, intense fatigue, difficulty of swallowing, the difficulty of breathing, hiccups, or headache*.” |
| Algorithm 2 | ECPS at the -3 point of cut-off | As described in Tshomba et al. [1], the extended clinical prediction score (ECPS) is composed of seven clinical or epidemiological predictors, and the presence or absence of each predictor is associated with a score. These predictors include: fatigue (score 0, if present; -1, if absent); difficulty in swallowing (score +1, if present; +0, if absent); red eyes (score +1, if present; +0, if absent); gingival bleeding (score +4, if present; +0, if absent); hematemesis (score +2, if present; +0, if absent); confusion (score +1, if present; +0, if absent); hemoptysis (score -2, if present; +0, if absent); and history of contact with an EVD case (score +2, if present; -1, if absent);  *“For each suspect, the total score is summed, and a suspect with a total score less than or equal to -3 is considered negative; otherwise, he is positive for the screening test.”* |
| Algorithm 3 | ECPS as a joint test | Using the ECPS tool in suspects whose level of exposition is known and categorized as follows:   - No risk exposure if there has been no reported EVD risk exposure. - Low-risk exposure, for instance, is any suspect who had direct contact with a live EVD patient and did not exhibit diarrhea, vomiting, or bleeding at the time of contact; any suspect who had touched patient clothes or sheets that were not obviously soiled; any attendance at a funeral or hospital visit but without reported direct contact. - Intermediate-risk exposure: Any interaction with a survivor of EVD who was vomiting, drooling, or bleeding, as well as any coming into contact with clothing or bedding that had been contaminated by feces, vomit, or blood. - High-risk exposures include taking care of an EVD patient who is vomiting, drooling, or bleeding; cleaning a patient's room or clothes; handling patient waste; and any direct contact with blood, body fluids (vomit, urine, feces), tissues, or skin. Other high-risk exposures include processing blood or body fluids from an EVD patient without using protective gear and coming into direct contact with the dead body of an EVD patient.   "In using the joint test or approach, all suspects at low-, intermediate-, and high-risk reported exposure should be clinically examined, and only those with a predicted probability of EVD greater than 5% should be recommended for isolation, while those with no risk exposure are considered not to have the disease." |
| Algorithm 4 | ECPS as a conditional test | "In using the conditional test or approach, all suspects with high-risk reported exposure, irrespective of their predicted probability of the disease, and then suspects with low and intermediate reported exposure having an EVD-predicted probability greater than 5%, should be isolated, while those with no risk exposure are considered not to have the disease." |
| Algorithm 5 | WHO case definition for EVD suspect and QuickNavi™-Ebola RDT in sequence | "In using this algorithm, the WHO case definition for EVD suspect is applied first to screen EVD, and then the QuickNavi™-Ebola RDT is applied to those who do not fit the WHO case definition for EVD suspect." |
| Algorithm 6 | ECPS at the -3 point of cut-off and QuickNavi™-Ebola RDT in sequence | "In using this algorithm, the ECPS at the -3 point of cut-off is applied first to screen EVD, and then the QuickNavi™-Ebola RDT is applied to those who are negative to the ECPS at the -3 point of cut-off." |
| Algorithm 7 | ECPS as a conditional test and QuickNavi™-Ebola RDT in sequence | "In using this algorithm, the ECPS as a conditional test is applied first to screen EVD, and then the QuickNavi™-Ebola RDT is applied to those who are negative to the ECPS as a conditional test." |
| Algorithm 8 | ECPS as a joint test and QuickNavi™-Ebola RDT in sequence | "In using this algorithm, the ECPS as a joint test is applied first to screen EVD, and then the QuickNavi™-Ebola RDT is applied to those who are negative to the ECPS as a joint test." |

**Reference**

1. Tshomba AO, Mukadi-Bamuleka DR, De Weggheleire A, Tshiani OM, Kitenge

RO, Kayembe CT, et al. Development of Ebola virus disease prediction scores: Screening tools for Ebola suspects at the triage-point during an outbreak. PLoS One. 2022;17(12):e0278678. Epub 20221216. doi: 10.1371/journal.pone.0278678. PubMed PMID: 36525443; PubMed Central PMCID: PMCPMC9757576.
