# Supplementary material for: Cost-effectiveness of incorporating Ebola prediction score tools and rapid diagnostic tests into a screening algorithm: A decision analytic model
Source: PLoS One. 2023 Oct 17;18(10):e0293077. doi: 10.1371/journal.pone.0293077 (PMC10581462; doi:10.1371/journal.pone.0293077)
Supplement: S2 File — (DOCX) [file pone.0293077.s002.DOCX]

**Supplementary appendix**

Formulas used to compute cost and effectiveness payoffs, and outcomes probability.

This is a detailed complement to “Materials and Methods” section which describes how cost-effectiveness and probabilities were calculated.

We computed the efficiency of each screening algorithm using the formula as follows:

$\boldsymbol{Efficiency of a screening algorithm}=\frac{Cost per an EVD suspect case screened}{Number of EVD case Isolated}$ (1)

We calculated the cost per an EVD suspect screened as follows:

$\boldsymbol{Cost per an EVD suspect case isolated}=[(total cost of outbreak surveillance)/(number of suspect screened)]+[(cost of SoC+cost of confirmation test+prime for lab technician)\times probability to isolate a true EVD)]+[(cost of SoC+cost of confirmation test+prime of laboratory technician)\times probability to isolate a non EVD)\times\left( 1-\left( 1 - \theta_{h} \right)ᵟ \right)\times average number of non\_EVD cases exposed in isolation because of FP]+[(cost of ruled out suspect care+(cost of SoC+cost of confirmation test+prime for lab technician)\times probability to isolate a true EVD\times R_{oc}\times(probabilty to rule out a false negative EVD)]+[(cost of ruled out suspect care )\times(probability to rule out a true negative EVD)]$ (2)

We computed the number of cases isolated as follows:

$\boldsymbol{Number of EVD cases isolated}=[({Prev}_{EVD} in suspeced people)\times(sensitivity of screening test sequence)\times effectiveness of EVD isolated) -(1-{Prev}_{EVD} in suspeced people)\times(probability of iatrogenic EVD)]$ (3)

Considering two scenarios, one test used as algorithm to screen Ebola suspect or two tests used as presented on figure below:


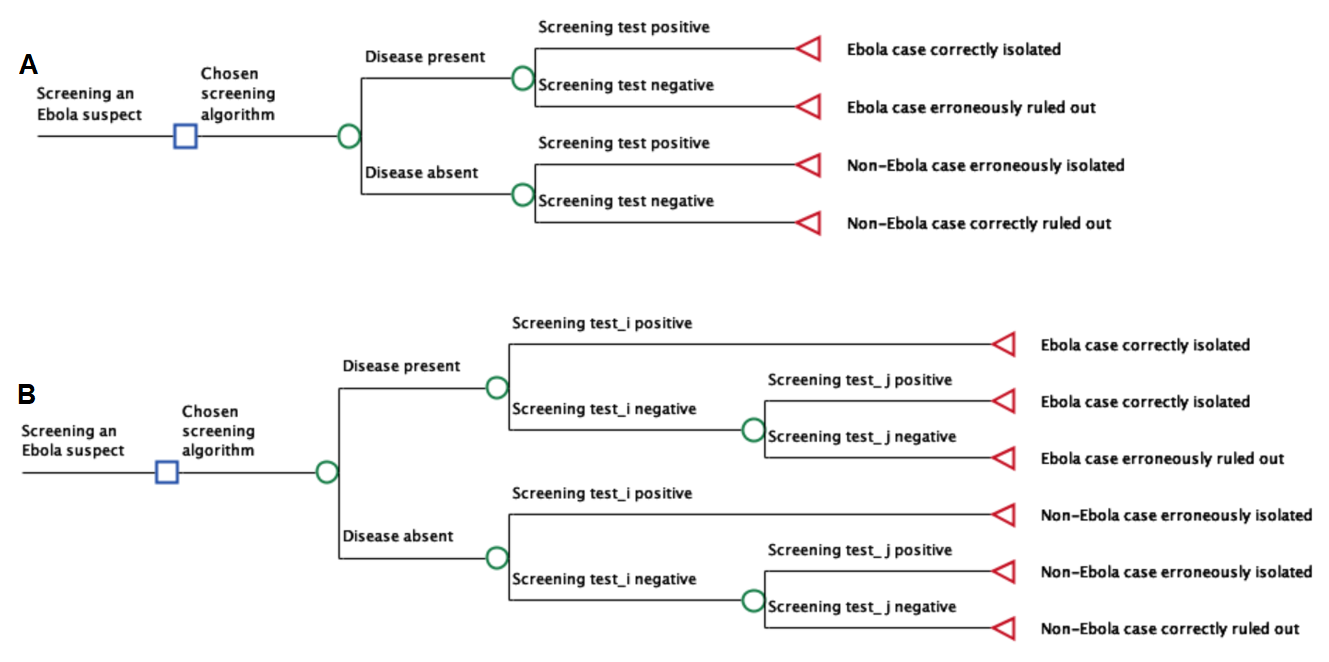


Where, i is a given first screening test applied and j, the second test applied, here RDT

Accordingly, we computed the expected outcome probabilities as follows:

1. For algorithm with one test used to screen suspects (scenario **A**)

$\boldsymbol{Probability to isolate an EVD}=\left[ {Prev}_{EVD}\times Sens_{t_{i}} \right]$ (4)

$\boldsymbol{Probability to isolate a non EVD}=\left[ \left( 1-{Prev}_{EVD} \right)\times\left( 1-Spe{c_{t}}_{i} \right) \right]$ (5)

$\boldsymbol{Probabilty to rule out an EVD}=\left[ {Prev}_{EVD}\times\left( 1- Sens_{t_{i}} \right) \right]$ *(6)*

$\boldsymbol{Probability to rule out a non EVD}=\left[ \left( 1-{Prev}_{EVD} \right)\times Spe{c_{t}}_{i} \right]$ (7)

1. For algorithm with two tests used to screen Ebola suspects (scenario B), tests in series

$\boldsymbol{Probability to isolate an EVD}=\left[ {Prev}_{EVD}\times(Sens_{t_{i}}+(1-Sens_{t_{i}})\times Sens_{t_{j}}) \right]$ (8)

$\boldsymbol{Probability to isolate a non EVD}=\left[ \left( 1-{Prev}_{EVD} \right)\times\left( \left( 1-Spe{c_{t}}_{i} \right)+Spe{c_{t}}_{i}\times\left( 1-Spe{c_{t}}_{j} \right) \right) \right]$ (9)

$\boldsymbol{Probabilty to rule out an EVD}=\left[ {Prev}_{EVD}\times\left( 1- Sens_{t_{i}} \right)\times\left( 1-Sens_{t_{j}} \right) \right]$ *(10)*

$\boldsymbol{Probability to rule out a non EVD}=\left[ \left( 1-{Prev}_{EVD} \right)\times Spe{c_{t}}_{i}\times Spe{c_{t}}_{j} \right]$ (11)

The probability of iatrogenic EVD is the probability to isolate a non-EVD multiplied by the probability of infection given random contact with an EVD patient.

The last probability was calculated using the formula used by Gilbert [1].

Thus, the probability of iatrogenic EVD is as follows:

For the algorithm with one screening test

$\boldsymbol{Probability of iatrogenic EVD}=\left[ \left( 1-{Prev}_{EVD} \right)\times\left( 1-Spe{c_{t}}_{i} \right)\times\left( 1-(1 - \theta_{h})ᵟ \right) \right]$ (12)

For algorithm with two tests

$\boldsymbol{Probability of iatrogenic EVD}=\left[ \left( 1-{Prev}_{EVD} \right)\times\left( \left( 1-Spe{c_{t}}_{i} \right)+Spe{c_{t}}_{i}\times\left( 1-Spe{c_{t}}_{j} \right) \right)\times\left( 1-(1 - \theta_{h})ᵟ \right) \right]$ (13)

Where $\theta_{h}$ is the Ebola secondary attack rate in the hospital setting and δ is one divided by the infectiousness duration.

In the same way, the probability that a ruled out EVD true case infect in the community, Ebola attack rate in the community is: 12.5%

For algorithms with one screening test:

$\boldsymbol{Probability of poor screening EVD}=\left[ {Prev}_{EVD}\times\left( 1- Sens_{t_{i}} \right)\times\left( 1-(1 - \theta_{c})ᵟ \right) \right]$ (14)

At last, for algorithms with two screening tests:

$\boldsymbol{Probability of poor screening EVD}=\left[ {Prev}_{EVD}\times\left( 1- Sens_{t_{i}} \right)\times\left( 1-Sens_{t_{j}} \right)\times\left( 1-(1 - \theta_{c})ᵟ \right) \right]$ (15)

Where $\theta_{c}$ is the Ebola secondary attack rate in the community and δ is one divided by the infectiousness duration.

As application in this paper:

- The Ebola secondary attack rate, $\theta_{h}$ is equals to 22.9%
- The infectiousness duration equals to 10 days,
- Thus, δ =1/Infectiousness duration for the EVD

=1/10

= 0.1

- Thus the probability of iatrogenic EVD is $\left( 1-(1 - \theta_{h})ᵟ \right)$ or (1-(1-0.229)^0.1^)=0.0257
- We assumed three non-EVD cases (FP and his two caregivers) would be exposed to Ebola infection in the isolation

Therefore, the iatrogenic payoff for a false positive is -0.0257$\times$3=0.077

- For the EVD erroneously ruled out in the community, the poor screening payoff for a false negative is minus the basic reproductive number (Ro), which accounts for the transmissibility and the typical number of community contacts that this false-negative would harm. The basic reproductive number was estimated at 2.49 by Lewnard [2].

Therefore, the poor screening harm due to false negative is -2.49

We gathered secondary attack rate and infectious duration from literature [3, 4]

# **References**

1. Gilbert JA, Meyers LA, Galvani AP, Townsend JP. Probabilistic uncertainty analysis of epidemiological modeling to guide public health intervention policy. Epidemics. 2014;6:37-45. Epub 20131119. doi: 10.1016/j.epidem.2013.11.002. PubMed PMID: 24593920; PubMed Central PMCID: PMCPMC4316830.

2. Lewnard JA, Ndeffo Mbah ML, Alfaro-Murillo JA, Altice FL, Bawo L, Nyenswah TG, et al. Dynamics and control of Ebola virus transmission in Montserrado, Liberia: a mathematical modelling analysis. Lancet Infect Dis. 2014;14(12):1189-95. Epub 20141023. doi: 10.1016/S1473-3099(14)70995-8. PubMed PMID: 25455986; PubMed Central PMCID: PMCPMC4316822.

3. Dean NE, Halloran ME, Yang Y, Longini IM. Transmissibility and Pathogenicity of Ebola Virus: A Systematic Review and Meta-analysis of Household Secondary Attack Rate and Asymptomatic Infection. Clin Infect Dis. 2016;62(10):1277-86. Epub 20160229. doi: 10.1093/cid/ciw114. PubMed PMID: 26932131; PubMed Central PMCID: PMCPMC4845791.

4. Rosello A, Mossoko M, Flasche S, Van Hoek AJ, Mbala P, Camacho A, et al. Ebola virus disease in the Democratic Republic of the Congo, 1976-2014. Elife. 2015;4. Epub 20151103. doi: 10.7554/eLife.09015. PubMed PMID: 26525597; PubMed Central PMCID: PMCPMC4629279.
