# Supplementary material for: Cost-effectiveness of incorporating Ebola prediction score tools and rapid diagnostic tests into a screening algorithm: A decision analytic model
Source: PLoS One. 2023 Oct 17;18(10):e0293077. doi: 10.1371/journal.pone.0293077 (PMC10581462; doi:10.1371/journal.pone.0293077)
Supplement: S1 Table — (DOCX) [file pone.0293077.s004.docx]

**S1 Table. Cost-effectiveness ratios (USD per EVD isolated) in relation to variation the cost of QuickNavi™-Ebola RDT and cost of standard-of-care (SOC)**

| **Cost of SOC** | **Algorithm** | **Cost-effectiveness of algorithms for a QuickNavi™-Ebola test cost (USD) of** | | | | | | | |
| --- | --- | --- | --- | --- | --- | --- | --- | --- | --- |
|  |  | **0** | **2.5** | **5** | **7.5** | **10** | **12.5** | **15** | **17.5** |
| 350 |  |  |  |  |  |  |  |  |  |
|  | 1 | 880.8 | 886.7 | 892.5 | 898.3 | 904.2 | 910.0 | 915.9 | 921.7 |
|  | 2 | 693.0 | 697.5 | 702.1 | 706.7 | 711.3 | 715.9 | 720.5 | 725.1 |
|  | 3 | 152.8 | 153.8 | 154.7 | 155.7 | 156.6 | 157.6 | 158.6 | 159.5 |
|  | 4 | 110.9 | 111.6 | 112.3 | 113.0 | 113.6 | 114.3 | 115.0 | 115.7 |
|  | 5 | 739.2 | 746.5 | 753.9 | 761.2 | 768.6 | 775.9 | 783.3 | 790.6 |
|  | 6 | 684.1 | 691.0 | 697.9 | 704.9 | 711.8 | 718.7 | 725.6 | 732.6 |
|  | 7 | 96.6 | 99.6 | 102.6 | 105.5 | 108.5 | 111.5 | 114.5 | 117.5 |
|  | 8 | 121.4 | 124.5 | 127.6 | 130.8 | 133.9 | 137.0 | 140.2 | 143.3 |
| 250 |  |  |  |  |  |  |  |  |  |
|  | 1 | 647.3 | 653.2 | 659.0 | 664.8 | 670.7 | 676.5 | 682.3 | 688.2 |
|  | 2 | 509.4 | 514.0 | 518.6 | 523.2 | 527.8 | 532.3 | 536.9 | 541.5 |
|  | 3 | 114.5 | 115.5 | 116.4 | 117.4 | 118.4 | 119.3 | 120.3 | 121.2 |
|  | 4 | 83.9 | 84.6 | 85.3 | 85.9 | 86.6 | 87.3 | 88.0 | 88.6 |
|  | 5 | 543.2 | 550.6 | 557.9 | 565.3 | 572.6 | 580.0 | 587.3 | 594.7 |
|  | 6 | 502.9 | 509.8 | 516.7 | 523.6 | 530.6 | 537.5 | 544.4 | 551.3 |
|  | 7 | 73.2 | 76.2 | 79.2 | 82.2 | 85.1 | 88.1 | 91.1 | 94.1 |
|  | 8 | 91.3 | 94.5 | 97.6 | 100.7 | 103.8 | 107.0 | 110.1 | 113.2 |
| 150 |  |  |  |  |  |  |  |  |  |
|  | 1 | 413.8 | 419.6 | 425.5 | 431.3 | 437.2 | 443 | 448.8 | 454.7 |
|  | 2 | 325.8 | 330.4 | 335 | 339.6 | 344.2 | 348.9 | 353.4 | 358.0 |
|  | 3 | 76.2 | 77.2 | 78.2 | 79.1 | 80.1 | 81.0 | 82.0 | 82.9 |
|  | 4 | 56.9 | 57.5 | 58.2 | 58.9 | 59.6 | 60.2 | 60.9 | 61.6 |
|  | 5 | 347.3 | 354.6 | 362.0 | 369.3 | 376.7 | 384.0 | 391.4 | 398.7 |
|  | 6 | 321.6 | 328.6 | 335.5 | 342.4 | 349.3 | 356.3 | 363.2 | 370.1 |
|  | 7 | 49.8 | 52.8 | 55.8 | 58.8 | 61.8 | 64.7 | 67.7 | 70.7 |
|  | 8 | 61.3 | 64.4 | 67.5 | 70.7 | 73.8 | 76.92 | 80.1 | 83.2 |
| 50 |  |  |  |  |  |  |  |  |  |
|  | 1 | 180.3 | 186.1 | 192 | 197.8 | 203.7 | 209.5 | 215.3 | 221.2 |
|  | 2 | 142.3 | 146.9 | 151.5 | 156.1 | 160.6 | 165.2 | 169.8 | 174.4 |
|  | 3 | 38.0 | 38.9 | 39.9 | 40.8 | 41.8 | 42.8 | 43.7 | 44.7 |
|  | 4 | 29.8 | 30.5 | 31.2 | 31.8 | 32.5 | 33.2 | 33.9 | 34.5 |
|  | 5 | 151.3 | 158.6 | 166.0 | 173.3 | 180.7 | 188.0 | 195.4 | 202.7 |
|  | 6 | 140.4 | 147.3 | 154.3 | 161.2 | 168.1 | 175.0 | 182.0 | 188.9 |
|  | 7 | 26.5 | 29.4 | 32.4 | 35.4 | 38.4 | 41.4 | 44.3 | 47.3 |
|  | 8 | 31.2 | 34.3 | 37.5 | 40.6 | 43.7 | 46.9 | 50.0 | 53.1 |
