# Supplementary figures and images for: Cost-effectiveness of incorporating Ebola prediction score tools and rapid diagnostic tests into a screening algorithm: A decision analytic model
Source: PLoS One. 2023 Oct 17;18(10):e0293077. doi: 10.1371/journal.pone.0293077 (PMC10581462; doi:10.1371/journal.pone.0293077)

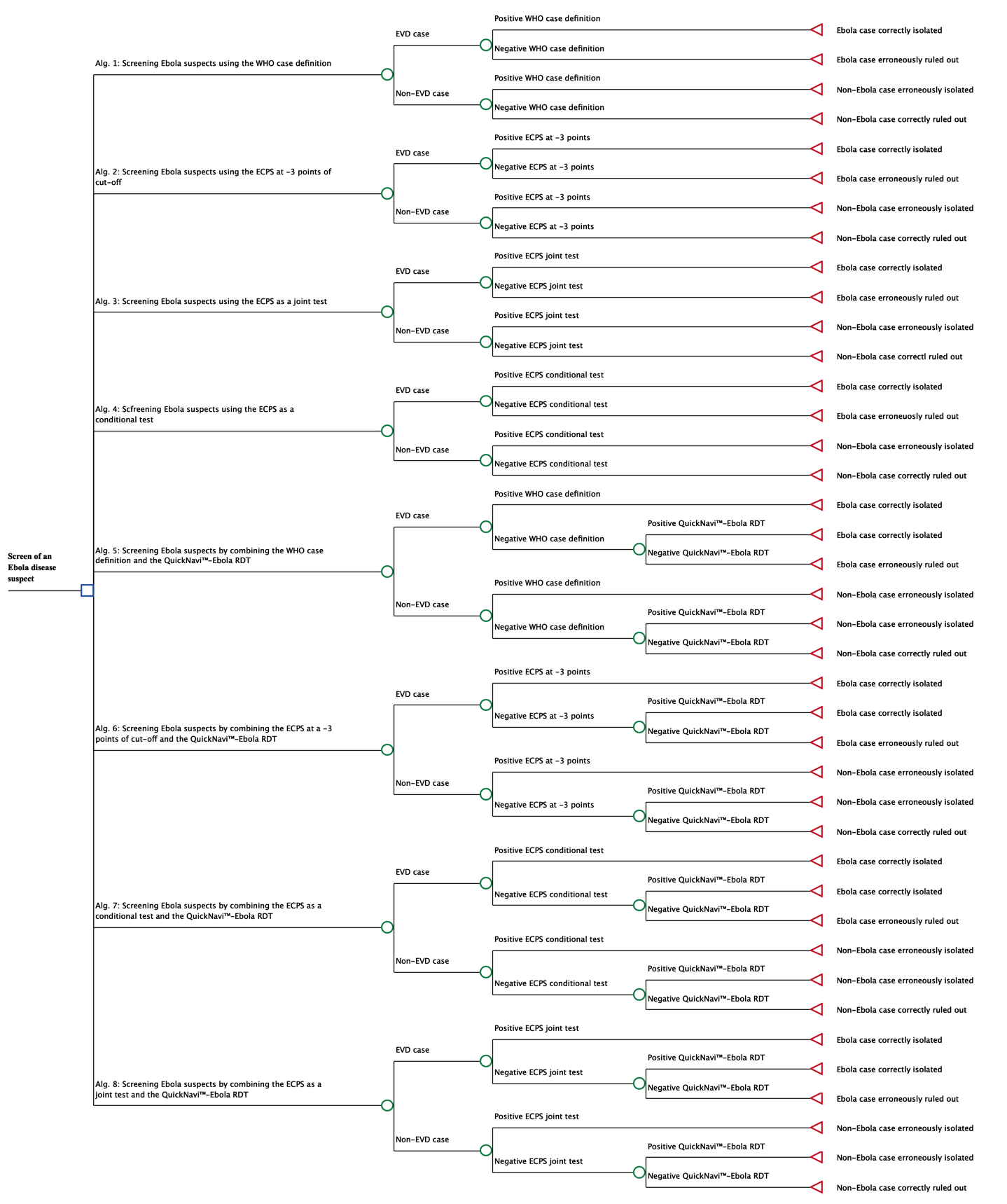

Supplement: S1 Fig — (TIF) [file pone.0293077.s005.tif]

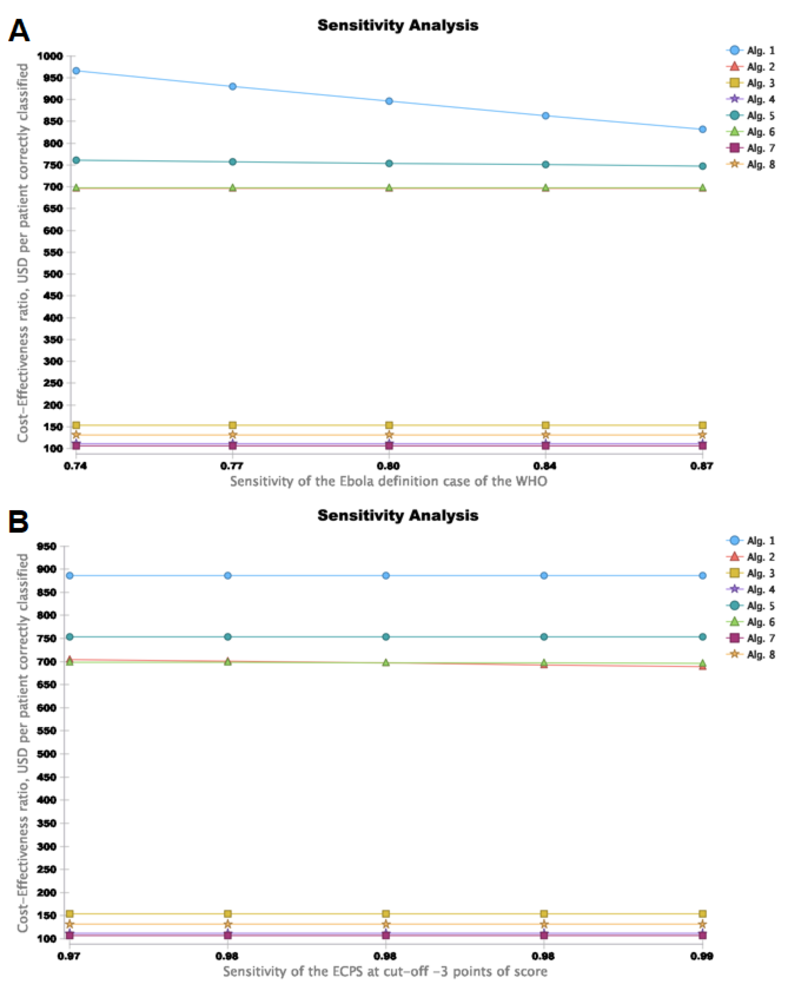

Supplement: S2 Fig — A is the effect of variation in the sensitivity of the WHO case definition for the suspect on the efficiency of algorithms. B is the effect of variation in the sensitivity of the ECPS at -3 points of cut-off on the efficiency of algorithms. (TIF) [file pone.0293077.s006.tif]

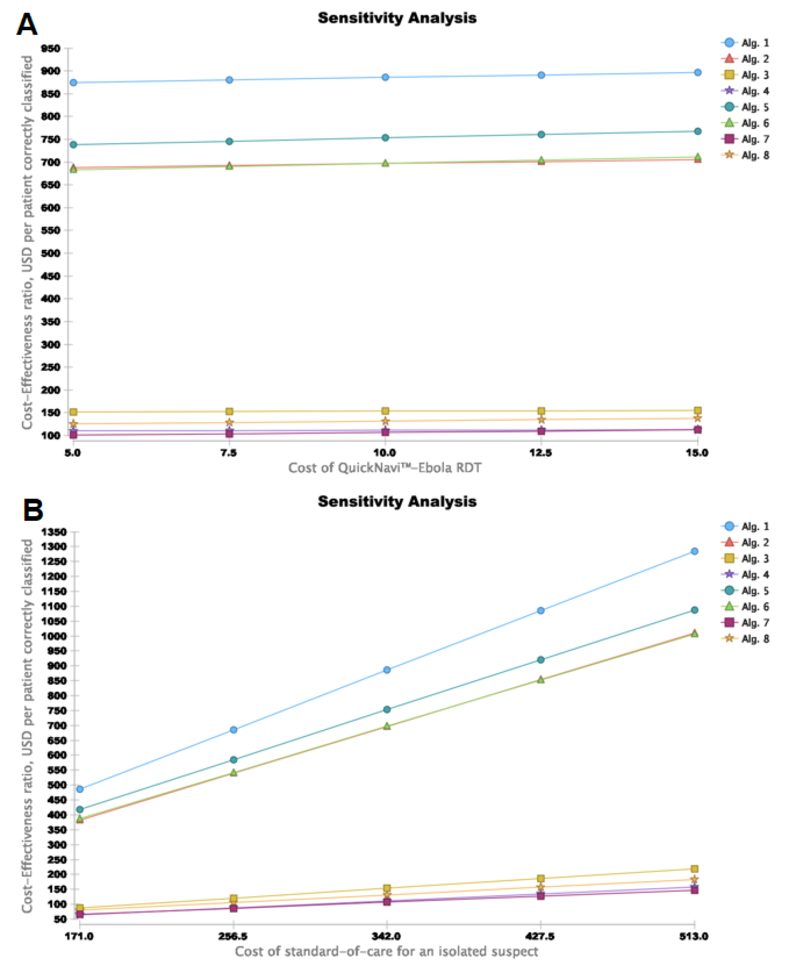

Supplement: S3 Fig — A presents the effect of variation in the cost of standard-of-care on the efficiency of the eight Ebola screening algorithms. B presents the effect of variation in the QuickNavi™-Ebola RDT cost on the efficiency of the 8 Ebola screening algorithms. (TIF) [file pone.0293077.s007.tif]

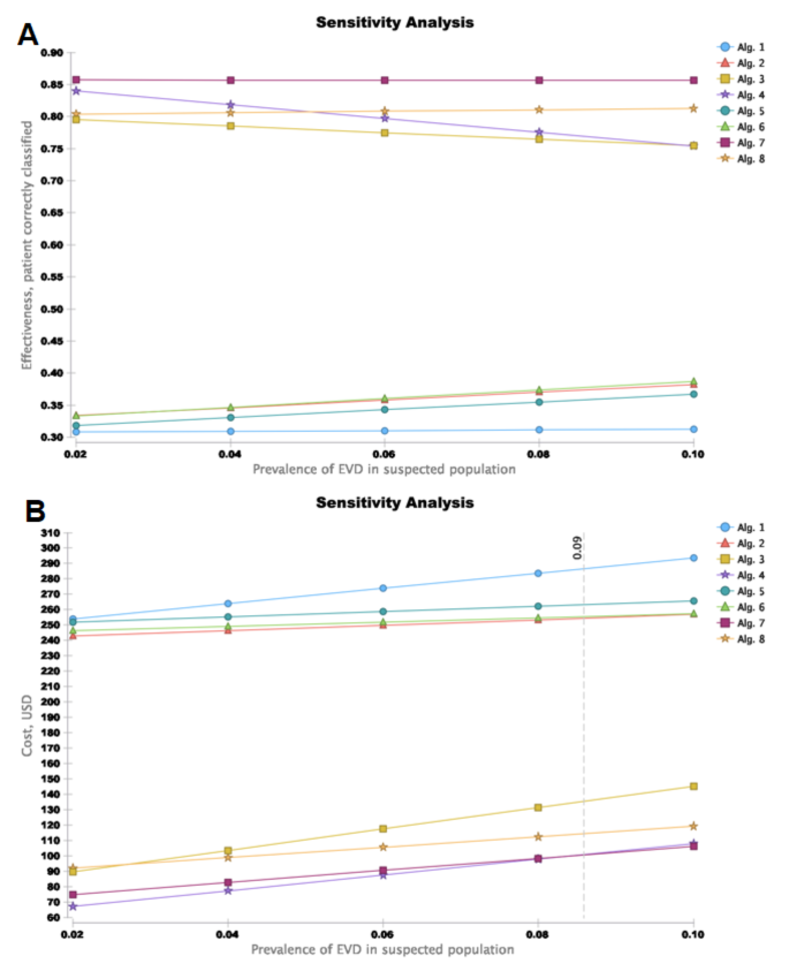

Supplement: S4 Fig — A depicts the effect of variation in the prevalence of Ebola virus disease on the effectiveness of screening algorithms. B, the effect of variation in the prevalence of Ebola virus on the cost of screening algorithms. The dotted horizontal line shows the threshold value of the prevalence over which the cost of the algorithm changes. Over this threshold of 10% of disease prevalence, the cost of ECPS as a joint or conditional test becomes low. Abbreviations: Alg. = algorithm; ECPS = extended clinical prediction score; EVD = Ebola virus disease. (TIF) [file pone.0293077.s008.tif]

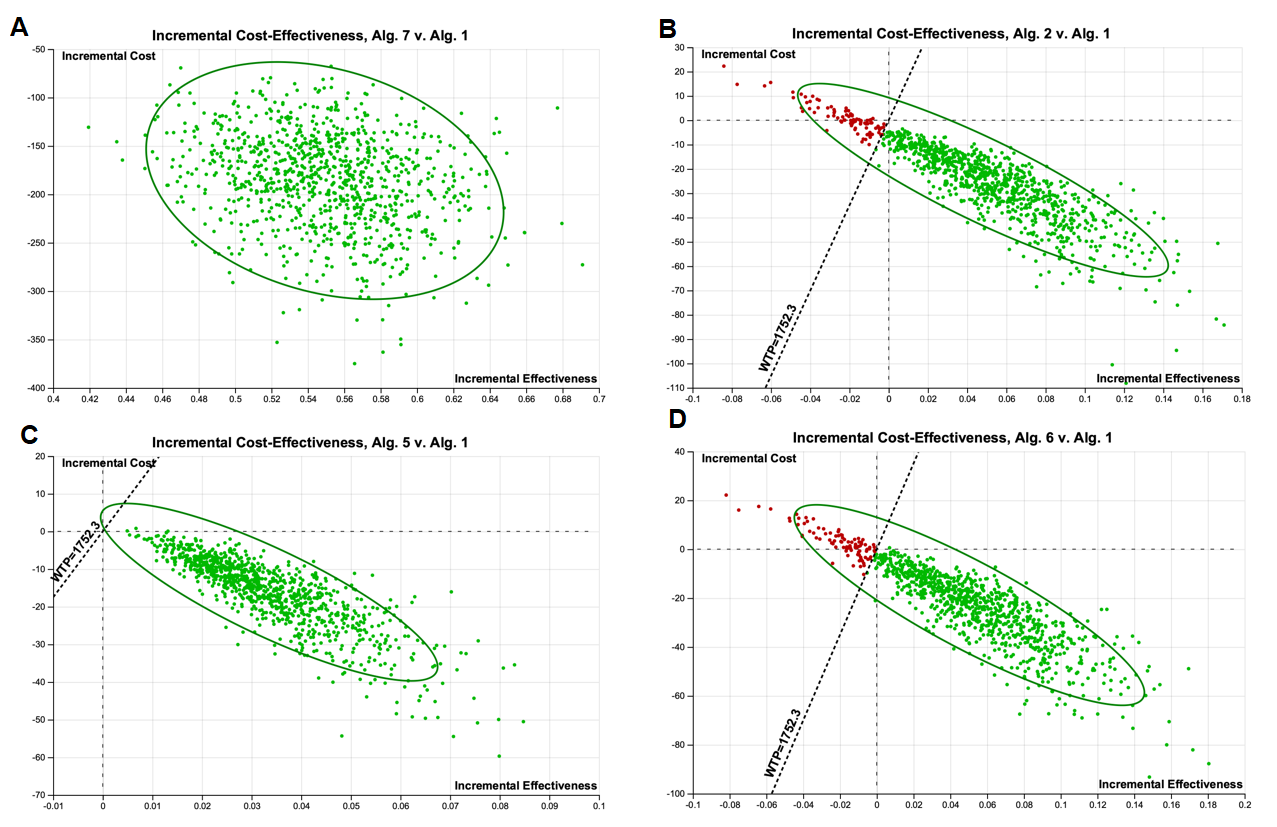

Supplement: S5 Fig — The ellipse represents 95% confidence points. The diagonal dashed line represents ICERs at a WTP threshold of USD 1,752.3. Points to the right of this dashed line are considered cost-effective. The dotted horizontal line shows an incremental cost of USD 0. Points below this line represent iterations in which an algorithm was cost saving compared with algorithm 1. This figure does not present all simulations of algorithms compared to algorithm 1. Those not presented here were cost- saving in 100% of simulations compared to algorithm 1 at this WTP threshold. Green points: ICERs that fall below the WTP line in Monte Carlo simulations, the maximum acceptable ICER (the algorithm is considered cost-effective); Red points: ICERs that fall above the WTP line, the maximum acceptable ICER (the algorithm is considered costly and less effective). Abbreviations: Alg. = algorithm; WTP = willingness to pay; ICER = incremental cost-effectiveness ratio. (TIF) [file pone.0293077.s009.tif]
