# Supplementary material for: Cost-effectiveness of incorporating Ebola prediction score tools and rapid diagnostic tests into a screening algorithm: A decision analytic model
Source: PLoS One. 2023 Oct 17;18(10):e0293077. doi: 10.1371/journal.pone.0293077 (PMC10581462; doi:10.1371/journal.pone.0293077)
Supplement: Attachment — Submitted filename: Response to Reviewers1.docx [file pone.0293077.s011.docx]

PONE-D-23-12291R1

Cost-effectiveness of incorporating Ebola prediction score tools and rapid diagnostic tests into a screening algorithm: a decision analytic model

PLOS ONE

Dear Dr. Tshomba,

Thank you for submitting your manuscript to PLOS ONE. After careful consideration, we feel that it has merit but does not fully meet PLOS ONE’s publication criteria as it currently stands. Therefore, we invite you to submit a revised version of the manuscript that addresses the points raised during the review process.

==============================
While the reviewer appreciate that most of the previous concerns were addressed, there are still several issues that need to be improved/addressed. Please revise your manuscript accordingly addressing all reviewer's comments.
==============================

Please submit your revised manuscript by Aug 16 2023 11:59PM. If you will need more time than this to complete your revisions, please reply to this message or contact the journal office at [plosone@plos.org](mailto:plosone@plos.org). When you're ready to submit your revision, log on to https://www.editorialmanager.com/pone/ and select the 'Submissions Needing Revision' folder to locate your manuscript file.

Please include the following items when submitting your revised manuscript:

- A rebuttal letter that responds to each point raised by the academic editor and reviewer(s). You should upload this letter as a separate file labeled 'Response to Reviewers'.
- A marked-up copy of your manuscript that highlights changes made to the original version. You should upload this as a separate file labeled 'Revised Manuscript with Track Changes'.
- An unmarked version of your revised paper without tracked changes. You should upload this as a separate file labeled 'Manuscript'.

If you would like to make changes to your financial disclosure, please include your updated statement in your cover letter. Guidelines for resubmitting your figure files are available below the reviewer comments at the end of this letter.

If applicable, we recommend that you deposit your laboratory protocols in protocols.io to enhance the reproducibility of your results. Protocols.io assigns your protocol its own identifier (DOI) so that it can be cited independently in the future. For instructions see: https://journals.plos.org/plosone/s/submission-guidelines#loc-laboratory-protocols. Additionally, PLOS ONE offers an option for publishing peer-reviewed Lab Protocol articles, which describe protocols hosted on protocols.io. Read more information on sharing protocols at https://plos.org/protocols?utm_medium=editorial-email&utm_source=authorletters&utm_campaign=protocols.

We look forward to receiving your revised manuscript.

Kind regards,

Jan Rychtář
Academic Editor
PLOS ONE

Additional Editor Comments:

While the reviewer appreciates that most of the previous concerns were addressed, there are still several issues that need to be improved/addressed.


[Note: HTML markup is below. Please do not edit.]

Reviewers' comments:

Reviewer's Responses to Questions

**Comments to the Author**

1. If the authors have adequately addressed your comments raised in a previous round of review and you feel that this manuscript is now acceptable for publication, you may indicate that here to bypass the “Comments to the Author” section, enter your conflict of interest statement in the “Confidential to Editor” section, and submit your "Accept" recommendation.

Reviewer #1: (No Response)

2. Is the manuscript technically sound, and do the data support the conclusions?

The manuscript must describe a technically sound piece of scientific research with data that supports the conclusions. Experiments must have been conducted rigorously, with appropriate controls, replication, and sample sizes. The conclusions must be drawn appropriately based on the data presented.

Reviewer #1: Partly

3. Has the statistical analysis been performed appropriately and rigorously?

Reviewer #1: Yes

4. Have the authors made all data underlying the findings in their manuscript fully available?

The PLOS Data policy requires authors to make all data underlying the findings described in their manuscript fully available without restriction, with rare exception (please refer to the Data Availability Statement in the manuscript PDF file). The data should be provided as part of the manuscript or its supporting information, or deposited to a public repository. For example, in addition to summary statistics, the data points behind means, medians and variance measures should be available. If there are restrictions on publicly sharing data—e.g. participant privacy or use of data from a third party—those must be specified.

Reviewer #1: Yes

5. Is the manuscript presented in an intelligible fashion and written in standard English?

PLOS ONE does not copyedit accepted manuscripts, so the language in submitted articles must be clear, correct, and unambiguous. Any typographical or grammatical errors should be corrected at revision, so please note any specific errors here.

Reviewer #1: Yes

6. Review Comments to the Author

Please use the space provided to explain your answers to the questions above. You may also include additional comments for the author, including concerns about dual publication, research ethics, or publication ethics. (Please upload your review as an attachment if it exceeds 20,000 characters)

Reviewer #1: Thank you for addressing the majority of my questions and comments. However, I still have concerns about how the costs of false positives and false negatives are being estimated, which were further exposed while I was reviewing the changes made during this revision. I think additional clarification and/or justification is required for the term of the form (1-(1-θ)^δ^) in equations 12-15 in S2 File, which estimates the “probability of infection given random contact with an EVD patient”. The secondary attack rate θ is estimated in the relevant reference (Okware et al) as the fraction of contacts of an EVD case that get infected. Given that definition, I don’t understand why the duration of infectiousness (1/δ) is being used here in this way. I suppose if you’re assuming that the risk to each suspected case entering isolation is equivalent to the risk of coming into contact with a single EVD patient for one day, and you are assuming the published secondary attack rate is being estimated from household contacts who are exposed continuously for the entire duration of infectiousness, there is some possible justification for this equation, which would need to be further explained in the manuscript. Unfortunately I believe that this risk estimate cannot be appropriately justified using this reasoning- it’s not clear from the reference for the secondary attack rate value exactly how a contact is defined, and there are published estimates for the household attack rate that are much higher than 2.5% (see the meta-analysis in Dean et al. Clin Infect Dis 2016 https://doi.org/10.1093/cid/ciw114 which includes the reference cited here). A better way to estimate infection risk for false positives would probably be to use results from a study specifically designed to measure the risk of nosocomial infection in patients erroneously isolated in EVD units (e.g., Arkell et al Tropical Medicine and International Health 2016
https://doi.org/10.1111/tmi.12802) which seem to estimate a higher risk of infection than calculated in this manuscript (though the absolute risk of exposure is still reasonably low).

The estimate of risk of community transmission from a false negative (which was calculated using the same reasoning) is even more problematic. Again, the assumption being made seems to be that the expected number of cases resulting from a true EVD case being allowed to remain in the community is equivalent to the risk of a single person coming into contact with that EVD patient for one day, except now the secondary attack rate is 50% lower than was estimated previously (presumably to account for lower risk from “asymptomatic” EVD patients). This seems to me to be quite an underestimation of the expected number of cases. First, I think these patients are not truly “asymptomatic” and instead may have nonspecific symptoms or be presymptomatic, so I don’t think there is good justification for the assumption that their infectiousness is lower (especially by an arbitrary 50%, which the authors do not justify). Also, I’m guessing that many of these patients will return to their homes after a negative screening, potentially exposing multiple people (possibly in higher-risk caregiving roles) over multiple days, making the expected number of new cases resulting from a single false negative to be much higher than the 0.00125 calculated here. Using the R0 value for EVD community transmission (while perhaps a bit of an overestimate) for the effect of a false negative is likely more correct, and more easily justified, and would result in a false negative penalty that is orders of magnitude higher than the value currently used here.

Since these false negative/positive risks are very important factors in the cost-effectiveness analysis (and thus the conclusions of the whole manuscript), they require suitable justification before I can recommend publication. I apologize for not noticing these issues in my first review.

**We appreciate your observation very much. We also appreciate the two informative documents you sent our way. We discovered further information in the articles that could offer trustworthy estimates of the expense and harm associated with false positives and false negatives.**

**According to the paper by Gilbert et al., this random probability (1-(1-θ)^δ) measures the risk of transmission during an infectious patient's entire period of time. We used this probability formula as such to compute the harm of errors in Ebola suspect-case classification. This probability is a function of both the secondary attack rate and the period or duration of infectiousness in an EVD patient.**

**Once again, thank you; we believed that the SAR values presented in Dean et al.'s work should be the ones we must employ. As a result, we used SAR estimates from the study by Dean et al. in place of the SAR by Okware et al.**

**For the false-positive isolated case, we did not employ the SAR for contacts providing nursing care because we believed that a frontline vaccination for healthcare professionals that is implemented in Ebola outbreak control nowadays in health setting would be applied to protect them from professional transmission. Therefore, since only isolated suspects who are not immunized would be exposed to this danger trough physical contact, we applied the direct physical contact SAR. As a result, we used the mean household SAR, or 22.9% (11.6%-34.2%), for those with direct contact but no nursing.**

**Similarly, for the false-negative case, we assumed that no nursing care is done for mild or asymptomatic Ebola cases ruled out for the community and that the conclusion from Mbala et al. and Leroy et al. is that mild or non-specific symptomatic Ebola has a reduced role in person-to-person transmission. Therefore, we used the overall household SAR, which is 12.5% (8.6%–16.3%).**

**Using these estimates, we were able to re-estimate the arm related to errors of classification, e.g., false positive and false negative harm.**

**For the random probability formula's parameter, for instance, (1-(1-θ) ^δ), which is defined as 1/ the length of time that an EVD is infectious (1 divided by the length of time that an EVD is contagious). This detail is not clear in the supplemental appendix S2. Therefore, we should make this explicit in the supplemental appendix S2 file by clarifying each part in Eq. 12 to 15.**

**Moreover, we did not include/use the R0 in our economic model. R0 is the number of secondary cases that one case would cause in a population that is completely susceptible, and it is estimated using biological, socio-behavioral, and environmental parameters that are involved in the transmission of the pathogen. As a result, R0 is calculated using ever-more complicated mathematical models, leading to incorrect interpretations and representations of R0 because each modeler develops models for specific objectives. Therefore, setting R0 numbers frequently given in the literature for historical epidemics may not be applicable to all Ebola outbreaks.**

**Additionally, R0 is one of the measures that is most frequently used to analyze the dynamics of infectious diseases, which was not the goal of this work.**

**We thus found it intuitive, simple to understand, and simple to generalize the random probability of contamination/transmission for false negatives (in the community) and false positives (in the healthcare setting) as a proportion of iatrogenic cases that could be generated in order to compute the errors of classification harm.**

**Therefore, we added these statements:**

**Method part of manuscript, on lines 221 to 224**

**As a frontline vaccination for healthcare workers would be implemented, we computed this probability of infection using the secondary attack rate (SAR) for direct physical contact of 22.9% (95% CI: 11.6%–34.2%) for those with direct contact but no nursing in the hospital (Ref. Dean et al.).**

**On lines 230 to 233**

**Therefore, we calculated the random probability that these false-negative cases generate Ebola cases in the community using the overall household SAR of 12.5% (95% CI: 8.6%–16.3 percentage) for human-to-human transmission in this setting without nursing (Ref. Dean et al**.).

**We corrected the harm associated with false positive and false negative in table 2 and in text.**

**In the results part of the manuscript:**

**We updated where needed accordingly (Tables 4 and 5, Figures 4 to 8, Supplementary Figures and the text of the manuscript**

I have a couple additional minor comments:

- I think Eq. 13 in S2 File is missing a 1-Prev term?

**Thank you very much. Yes, this term is missing in this formula. Thus, we corrected that as follows:**

$$\boldsymbol{Probability of iatrogenic EVD}=\left[ \left( 1-{Prev}_{EVD} \right)\times\left( \left( 1-Spe{c_{t}}_{i} \right)+Spe{c_{t}}_{i}\times\left( 1-Spe{c_{t}}_{j} \right) \right)\times\left( 1-(1 - \theta_{h})ᵟ \right) \right]$$

- Please explicitly state in the data availability statement that the raw data are available in the Supporting Outputs Data so readers know exactly where to find them.

**Thank you very much.**

**Added on line 657 of data availability statement:**

**in supporting information-Compressed file (the Supporting Outputs Data).**

- Figs. 7 and 8 legends: define red/green colors of points

**Thank you very for this observation.**

**We added in Fig. 7 and 8 legends and Suppl. Fig. legends these statements:**

**Green points: ICERs that fall below the WTP line in Monte Carlo simulations, the maximum acceptable ICER (the algorithm is considered cost-effective); Red points: ICERs that fall above the WTP line, the maximum acceptable ICER (the algorithm is considered costly and less effective).**

- It’s still a little unclear in the text what “joint” and “conditional” mean when referring to the ECPS test. The authors define these terms very clearly in their previous publication (ref. [26] in this manuscript, relevant passage copied below), and I’d suggest adding a similar description here or at least referencing this publication at the point in the text where these algorithms are defined.

“Finally, we evaluated our prediction models according to two additional clinical practice approaches in healthcare settings: joint and conditional tests or approaches. In both approaches, the suspects with no reported risk of exposure would be considered to not have the disease and the clinical team would act accordingly. No additional action, e.g., isolation, would be required. In the joint approach, the clinical team should clinically examine all suspects at low-, intermediate-, and high-risk reported exposure and recommend for isolation only those with a predicted probability of EVD greater than 5% (the cut-off chosen to maximize sensitivity, about 90 percent, in disease adverse context). In the conditional approach, the clinical team should isolate all suspects with high-risk reported exposure irrespective of their predicted probability of the disease and then suspects at low and intermediate reported exposure having an EVD-predicted probability greater than 5%.”

**Thank you for this observation. This could make clear their use. Thank you.**

**We added these statements:**

**Method part of the manuscript, on lines 155 to 164:**

**As described by Tshomba et al., the two screening methods—joint and conditional tests with ECPS—are methods in which suspects with no reported risk of exposure would be assumed to be free of the disease, and the clinical team would act appropriately (e.g., no further action is taken). In using the joint approach, all suspects at low-, intermediate-, and high-risk reported exposure are clinically assessed, and only those with a predicted likelihood of EVD greater than 5% are suggested for isolation. In the conditional test, regardless of their estimated probability of contracting the illness, all suspects with high-risk reported exposure should be isolated. Next, suspects with low and intermediate reported exposure who have an EVD-predicted probability of more than 5% should be isolated.**

7. PLOS authors have the option to publish the peer review history of their article (what does this mean?). If published, this will include your full peer review and any attached files.


If you choose “no”, your identity will remain anonymous but your review may still be made public.


**Do you want your identity to be public for this peer review?** For information about this choice, including consent withdrawal, please see our Privacy Policy.

Reviewer #1: **Yes:** Debra Van Egeren

[NOTE: If reviewer comments were submitted as an attachment file, they will be attached to this email and accessible via the submission site. Please log into your account, locate the manuscript record, and check for the action link "View Attachments". If this link does not appear, there are no attachment files.]

While revising your submission, please upload your figure files to the Preflight Analysis and Conversion Engine (PACE) digital diagnostic tool, https://pacev2.apexcovantage.com/. PACE helps ensure that figures meet PLOS requirements. To use PACE, you must first register as a user. Registration is free. Then, login and navigate to the UPLOAD tab, where you will find detailed instructions on how to use the tool. If you encounter any issues or have any questions when using PACE, please email PLOS at figures@plos.org. Please note that Supporting Information files do not need this step.

**Thank you. We analyzed them using PACE.**
